# Post-COVID-19 Symptoms in Adults with Asthma—Systematic Review

**DOI:** 10.3390/biomedicines11082268

**Published:** 2023-08-14

**Authors:** Marek Kaszuba, Natalia Madej, Rafal Pilinski, Agnieszka Sliwka

**Affiliations:** 1 Unit of Rehabilitation in Internal Diseases, Institute of Physiotherapy, Faculty of Health Sciences, Jagiellonian University Medical College, 31-066 Kraków, Poland; marek.kaszuba@uj.edu.pl (M.K.); rafal.pilinski@uj.edu.pl (R.P.); 2 Doctoral School in Medical and Health Sciences, Jagiellonian University Medical College, 31-530 Kraków, Poland; natalia.madej@doctoral.uj.edu.pl

**Keywords:** post-COVID-19 symptoms, long COVID, asthma, systematic review

## Abstract

Background: Research on the longer-term sequelae of COVID-19 in patients with asthma is limited. Objective: To assess the frequency and severity of long-term symptoms of COVID-19 in the population of asthma patients. Methods: A systematic review of the published literature was conducted in accordance with the recommendations of the PRISMA statement. EMBASE, MEDLINE/PubMed, Web of Science, CINAHL, and Scopus Scholar were searched for terms related to asthma and post or long COVID-19, and for systematic reviews related to specific questions within our review, up to June 2022. Results: Data from 9 references publications included in the review were extracted. A total of 1466 adult asthmatic patients with COVID-19 infection were described in all the publications mentioned above. Of the long-term symptoms reported after COVID-19, patients indicated: lower respiratory symptoms, fatigue, cognitive symptoms, psychological problems, and other such as skin rashes, gastrointestinal disorders, tachycardia, palpitations, ocular disorders, ageusia/hypogeusia, anosmia/hyposmia, and poor sleep quality. These symptoms in similar intensity were observed in the comparison groups without a diagnosis of asthma. Conclusions: The published data neither confirm nor deny that long-term COVID-19 symptoms in patients with asthma diagnosis are different in strength and frequency from patients without asthma diagnosis. To indicate associations between asthma and COVID-19 infection and severity, as well as the frequency of long-term symptoms of COVID-19, more longitudinal research is needed in chronic asthma patients with different phenotypes, intensity of treatment, and degree of asthma control.

## 1. Introduction

According to the recent update of the Global Initiative for Asthma (GINA), asthma is a heterogeneous disease, usually characterized by chronic inflammation of the airways. In the medical history, there are respiratory symptoms such as wheezing, chest tightness, cough that varies with time and intensity, shortness of breath, and limitation of variable expiratory airflow (Global Initiative for Asthma. Global Asthma Management and Prevention 2022; Available at: www.ginasthma.org [1]. Patients with asthma are at increased risk of developing exacerbations after being infected with respiratory viruses such as the influenza virus, parainfluenza virus, and severe acute respiratory syndrome coronaviruses (SARS-CoV) [2].

During the months after the beginning of the COVID-19 pandemic, studies were conducted to understand the relationship between asthma and the risk of SARS-CoV-2 infection, the severity, and the prognosis associated with the disease [3]. The task was not easy, as the reported prevalence of COVID-19 in people with asthma was varied in different parts of the world [4,5,6,7]. Studies, which were the source of knowledge, included asthmatic populations with various methods of asthma diagnosis and severity, different asthma phenotypes (allergic or non-allergic) and different asthma medications (corticosteroids treated or corticosteroids naive patients, biologics), other comorbidities, which can explain the different levels of susceptibility and severity of COVID-19 [3,8].

A recent GINA update on asthma and COVID-19 concluded that, according to currently available evidence, people with well-controlled, mild to moderate asthma do not appear to have an increased risk of developing COVID-19 and developing severe COVID-19; however, the risk of death from COVID-19 is increased in people who had recently needed oral corticosteroids (OCS) for their asthma and in hospitalized patients with severe asthma [1]. Although lower respiratory infections may worsen asthma control, the disease control (measured by ACT), based on available data, seems to be unaffected by COVID-19, at least in most patients, in terms of long-term perspective [2,9].

In a systematic review and meta-analysis of 131 studies, Liu et al. suggested that asthma was not associated with more severe phenotypes of COVID-19 and poor results [10]. Furthermore, patients with asthma did not show an increase in the need for intubation/mechanical ventilation compared to patients without asthma. An update published in 2021 concludes that asthma is not overrepresented in hospitalized patients with severe pneumonia due to SARS-CoV-2 infection and that there was no increased risk of exacerbations of asthma caused by the virus. However, asthma phenotypes and comorbidities are claimed to be important factors in assessing the risk of SARS-CoV-2 infection and disease [3]. The large population-based cohort study of almost 500,000 UK citizens demonstrated that adults with asthma had a higher risk of severe COVID-19, which was driven by the increased risk in patients with nonallergic asthma [11]. Likewise, adults with severe asthma are more likely to be hospitalized for COVID-19. It would be important to see if severe asthma increases the risk of long-COVID-19 after SARS-CoV-2 infection [12]. Angiotensin converting enzyme-2 (ACE2) and the enzyme proteases are needed for viral attachment and invasion into host cells. Levels of these enzymes may differ by clinical or molecular phenotypes of asthma. A study reported that asthma with high allergic sensitization was associated with low expression of ACE2, contrary to neutrophilic inflammation that is strongly associated with sputum FURIN expression levels, which can indicate the potential for a greater morbidity and mortality outcome from SARS-CoV-2 infection in neutrophilic severe asthma [13,14]. Such reassuring results of the first summaries and systematic reviews have come into question in the face of the increasingly recognized late phase of COVID-19 (now widely known as long COVID, or the post-COVID-19 condition). It has been reported in patients admitted to hospital with severe COVID-19, as well as in children and adults who initially experienced mild disease [15]. There is no single popularized definition of the so-called post-COVID-19 syndrome in the literature. A review of more than 120 primary articles on the subject [16] points to the wide variety of clinical manifestations referred to this term. The National Institute of Health and Care Excellence (NICE) defined the “Post-COVID-19 syndrome” as “signs and symptoms that develop during/after COVID-19 infection that persist for more than 12 weeks and could not be explained by any other diagnosis” [17]. These persistent symptoms could be the result of residual inflammation, organ damage, nonspecific effects of hospitalization or prolonged ventilation, social isolation, or impact on pre-existing health conditions [18]. The most prevalent long-COVID-19 signs/symptoms reported in a previous systematic review are chest pain (up to 89%), fatigue (up to 65%), dyspnoea (up to 61%), cough and sputum production (up to 59%), cognitive and memory impairment (up to 57.1%), arthralgia (up to 54.7%), sleep disorders (up to 53%), myalgia (up to 50.6%), and functional impairment (up to 50%) [19].

Recent reports also point to the risk of long-term sequela with cutaneous, cardiovascular, musculoskeletal, neurologic, and renal involvement in those who survive the acute phase of the disease [16].

Looking for a profile of patients who are most often found to have post-COVID-19 syndrome, the authors of a large observational study identified an association of age between 40 and 60 years, hospital admission at the beginning of symptoms, severe COVID-19, and dyspnoea or abnormal chest auscultation with post-COVID-19 syndrome [20]. It was also reported that those with more than five symptoms in the first week of the acute phase of COVID-19 were four times more likely to develop the post-COVID-19 syndrome, especially among women, older people, and those with obesity [13,21].

This raises the question whether, in patients with preexisting asthma, a lower susceptibility to COVID-19 also affects the long-term persistence of disease symptoms. The first single-centre reports on the subject have already appeared in the literature [22]. Recently published international consensus on research priorities for patients with preexisting airway disease in terms of long-term sequelae of COVID-19 indicated that the effect of COVID-19 on patients with preexisting airway disease should be prospectively studied in large cohorts of patients [23]. That is why we set out to review and summarize existing publications to answer the following questions.

### Objective/Review Questions

The purpose of the systematic review was to answer the following questions.

Primary questions:

1. What are the long-term symptoms (lasting at least 12 weeks) caused by SARS-CoV-2 infection among patients with asthma?
2. Are the long-term symptoms (lasting at least 12 weeks) caused by SARS-CoV-2 in patients diagnosed with asthma different from those of patients without asthma?

Secondary questions:

1. What does the evidence tell us about the long-term consequences for lung function, asthma symptoms, intensity of pharmacological treatment, physical fitness, and mental health of adults with asthma after SARS-CoV-2 infection?
2. Does asthma control before SARS-CoV-2 infection and asthma phenotype influence the severity of the disease, lung function, and the severity of long-term symptoms after infection?

## 2. Methodology

The review was carried out according to the Preferred Reporting Items for Systematic Reviews and Meta-Analyses (PRISMA). The review protocol was registered in the PROSPERO database with the number CRD42021242960 (Centre for Reviews and Dissemination, University of York, York, UK).

### 2.1. Searches

A systematic search of electronic databases: EMBASE, MEDLINE/PubMed, Web of Science, CINAHL, Scopus were done for articles published in English from 1 January 2020 to 30 June 2022. 

The search strategies were adapted to each database and used a combination of terms related to or describing SARS-CoV-2; post-COVID-19 symptoms; long-COVID-19 symptoms; and asthma. A detailed combination of words and terms is presented in Table S1. 

Following the primary searches, manual searching of the citations and reference lists of the included manuscripts was undertaken to ensure completeness. 

### 2.2. Types of Included Studies 

Inclusion criteria:

Studies involving long-term consequences of COVID-19 (present 12 weeks after the onset of the disease or longer), including epidemiological, laboratory, and clinical data evaluated in adults with asthma. Eligible studies could be observational (prevalence studies, cross-sectional studies) and interventional (cohort studies, randomized control trials, and case-control studies).

Exclusion criteria:

- Studies focusing on COVID-19 symptoms lasting <12 weeks from the onset of the disease or only on the pathophysiology of COVID-19 in asthma.
- Age of the participants <18 years.
- Studies without evidence of laboratory confirmation of SARS-CoV-2 infection in participants.
- Studies without information about asthma diagnosis in participants.
- Secondary analysis (i.e., systematic review or meta-analysis); however, their reference lists have been manually searched for potentially relevant studies.
- Literature in languages other than English.

### 2.3. The Condition Being Studied and the Population of Patients 

Long-term symptoms (lasting at least 12 weeks) in confirmed cases of COVID-19 (based on nucleic acid amplification tests, antigen testing, or serologic tests) with pre-existing asthma diagnosis.

### 2.4. Comparator(s)/Control

Patients with asthma have been compared to people with confirmed COVID–19 (based on nucleic acid amplification tests, antigen testing, or serologic tests) without asthma diagnosis. 

### 2.5. Main Outcome(s)

Long-term symptoms associated with COVID-19, their prevalence, and severity (if available) in patients with asthma compared to patients without asthma.

### 2.6. Additional Results

1. Pulmonary function of people with asthma 12 weeks after SARS-CoV-2 infection [forced vital capacity (FVC); forced expiratory volume in the first second (FEV1); FEV1/FVC ratio; total lung capacity (TLC), residual volume (RV), and vital capacity (VC)].
2. Severity of symptoms within 12 weeks after COVID-19 infection [dyspnoea: Modified Medical Research Council dyspnoea scale (mMRC), asthma control test (ACT), asthma control questionnaire (ACQ); Asthma Quality of Life Questionnaire for asthma (AQLQ)]; Depression scale, the number of cases with decreased asthma control after COVID-19.
3. Modification of the intensity of asthma pharmacological treatment after COVID-19 infection.

### 2.7. Data Extraction (Selection and Coding)

The study selection process has been done with the help of the systematic review software Covidence (Veritas Health Innovation, Melbourne, Australia) [24]. The titles and abstracts of all records (*n* = 3198) recovered during the searches were screened by two pairs of reviewers (MK, AS, NM, RP), after accounting for duplication of eligibility, against the inclusion/exclusion criteria. 

The full texts of all (*n* = 95) potentially relevant studies were examined to determine which should be included in the final selection of the study. 

**Figure 1:**
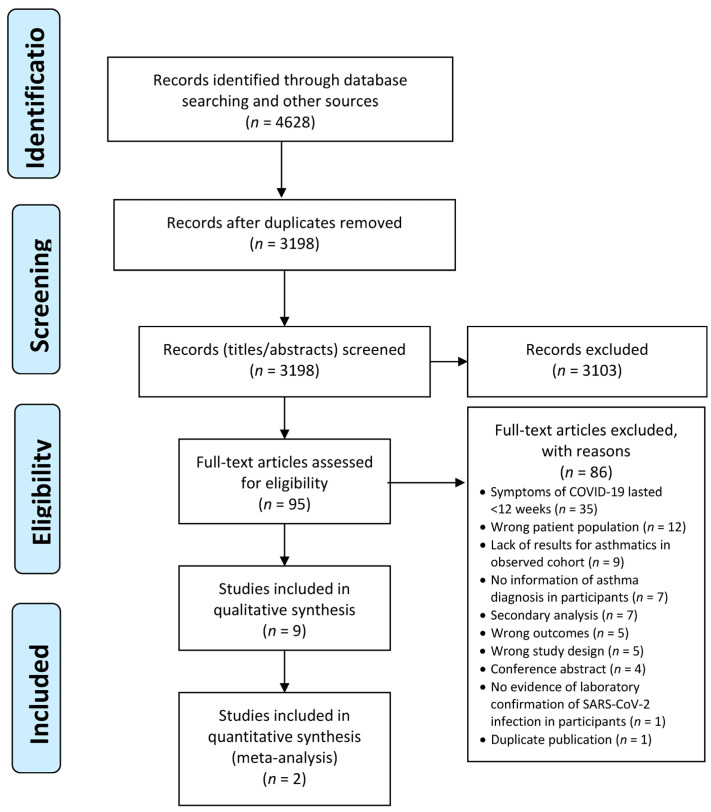
The PRISMA flow diagram.

Any discrepancies have been resolved in Covidence by a third reviewer (AS, MK). Studies (*n* = 85) that were excluded when obtaining the full text have been recorded, accompanied by a justification for the exclusion. A PRISMA flow diagram demonstrates the different phases of this process (Figure 1). 

Data were extracted from the 10 eligible studies by four authors and verified by another reviewer. As two publications described the same study, the data were extracted together, ultimately reducing the number of studies described to 9.

### 2.8. Evaluation of Risk of Bias (Quality)

The methodological quality of the included studies has been evaluated using Joanna Briggs Institute critical appraisal tools, which are adjusted to assess different types of primary studies [25]. The purpose of this evaluation is to help assess the trustworthiness, relevance, and results of the published papers by assessing their methodological quality, determining the extent to which a study has addressed the possibility of bias in its design, conduct, and analysis. The JBI critical evaluation tools have been developed by the JBI and its collaborators and approved by the JBI Scientific Committee after extensive peer review.

To properly apply the tool, the decision on whether a publication was evaluated using the observational cohort or cross-sectional study assessment tool was made on a case-by-case basis, depending on the phase of the study from which the data used in the review came from. If the data came only from the final observation stage of the cohort, it was more appropriate to assess the reliability of the publication using the cross-sectional assessment tool.

### 2.9. Strategy for Data Synthesis

The estimated prevalence of post-COVID-19 syndrome was pooled between studies using a random effects model. Analyses were performed using the Statistica version 13 software (StatSoft Polska, Krakow, Poland). The analyses in the subgroups were not carried out because of the lack of studies providing sufficient numerical data. Sensitivity analyses were planned to be performed using inverse variance heterogeneity models if heterogeneity, as defined by the I^2^ test, is found to be high. Following the sensitivity analysis, to obtain a homogeneity appropriate for meta-analysis, certain literature was to be deleted or subgroup analyses were to be conducted, as appropriate.

## 3. Results

We finally included seven studies published in 2021 [22,26,27,28,29,30,31] and two published in 2022 [32,33] (Table 1). Six of them were prospective cohort observations [22,27,28,30,31,32]; two were cross-sectional studies [29,33], and one was a case-control study [26]. Most studies included patients with a confirmed diagnosis of SARS-CoV-2 infection, without narrowing this group to patients with asthma. From the described COVID-19 population, subgroups were then extracted based on other variables, including comorbidity. Thus, we obtained knowledge of potential long-COVID-19 symptoms in asthmatic patients in eight out of nine studies [22,26,27,28,29,30,32,33]. Only Foster et al. recruited exclusively asthmatic patients with COVID-19 infection for the study and observed symptoms that occurred 4–6 months after infection [31]. Most studies included only those with an objectively confirmed diagnosis of SARS-CoV-2 infection, by RT-PRC [22,26,27,28,29,30,31,32]. A study also included patients with a confirmed diagnosis by antigen test (Anti-SARS-CoV-2 immunoglobulin G titer) [27]. Finally, only one study allowed the participation of respondents who stated during the online survey that they had the disease, with or without test verification [33]. However, our analysis included data from this publication that referred only to participants with confirmed COVID-19, who accounted for 10.5% of all the study participants, which is broadly in line with UK SARS-CoV-2 antibody survey data from the same period [34].

Regarding the valid confirmation of asthma diagnosis, only four authors of included studies confirmed that they used the GINA criteria to diagnose and classify asthma patients in the observed patient populations [22,26,29,32]. In one paper, the author reports that patients self-reported that they had asthma, which, however, was not objectively verified [33]. Another of the included articles reports that the patient’s “medical history” was used to confirm the diagnosis of asthma [31]. Despite enquiries sent to the other authors, no response was received as to how and what type and stage of asthma was diagnosed. 

A total of 1466 asthmatic patients with COVID-19 infection were described in all the publications mentioned above. Of this number, the diagnosis of both conditions was correctly described in 848 individuals [22,26,29,32]. Of the symptoms referred to after COVID-19, patients with asthma mentioned: lower respiratory symptoms: dyspnoea during exertion (75.4%) and at rest (34.4%), cough (2.1%), moreover: fatigue (21.2–65.6%), memory loss (9.1–19.0%), skin rashes (10.2–13.1%), concentration loss (8.1–13.1%), cognitive twilight—brain fog (9.6–9.9%), tachycardia-palpitations (6.7–9.8%), gastrointestinal disorders—diarrhoea (3.3–7.2%), ocular disorders (4.5–6.6%), anosmia/hyposmia (3–6.6%), ageusia/hypogeusia (1.6–3.3%), throat pain (3.3%), depressive symptoms, anxiety symptoms, poor sleep quality. Some of them reported an increase in reliever inhaler use and that asthma management was worse or much worse. In one study there was no difference in the frequency of reported symptoms between the asthmatic and non-asthmatic groups [26]. Although Egger et al. reported no difference in time to resolution of symptoms between patients with asthma and without asthma [32], Pachon et al. stated that patients with pre-existing asthma have a lower prevalence of post-COVID-19 syndrome than that reported among the totality of patients with COVID-19 [22]. 

On the contrary, Munblit et al. showed that preexisting asthma was associated with a higher risk of neurological 1.95 (1.25–2.98) and mood and behavioural changes 2.02 (1.24–3.18) [28]. Neurological persistent symptoms included tingling feeling/“pins and needles“, fainting/blackouts, seizures/fits, tremor/shakiness, double vision, problems speaking or communicating, problems with balance, confusion/lack of concentration, dizziness/light headedness, and forgetfulness. Mood and behaviour persistent symptoms included anxiety, loss of interest or pleasure, behavioural [18] changes, depressed mood.

Of the objectively measured variables, only two papers presented the results of the measurements of the spirometry variables [27,30]. Although a study reported values for variables such as DLCOcSB, TLC, FVC, and FEV1 measured after a 4-month follow-up of COVID-19, the publication does not report the rough data obtained by the nine patients with asthma selected from the observed cohort [27]. In the second study, different results in this regard were presented, in which patients with asthma (*n* = 18) had higher odds of altered DLCO (OR = 4.86, 95% CI: 1.09; 21.68) [30]. In these studies, however, we noticed a significant limitation regarding the quantitative data from small spirometry samples, which did not allow us to draw consistent conclusions.

The most detailed description of the observed asthma group was provided by Garcia-Pachon et al., who included 74 people with asthma in their prospective cohort study [22]. Severity of asthma in this publication was classified according to the prescribed therapy following international GINA recommendations [1]: mild (steps 1 and 2 of GINA), moderate (steps 3 and 4), or severe (step 5). Asthma was severe in 5 (7%), moderate in 52 (70%), and mild in 17 (23%) patients (four receiving omalizumab and one benralizumab). Although Garcia-Pachon et al. did not describe the level of asthma control nor percentage of inhaled corticosteroid intake in the patients, it was the only study in which the authors determined the phenotype of asthma. Forty-six patients were classified as allergic asthma, seven as eosinophilic asthma, and twenty one as non-T2 asthma. Thirty-four patients (46%) developed symptoms but did not require hospital admission during SARS-CoV-2 infection, fifteen (20%) were hospitalized, and the rest were asymptomatic. Seven of the seventy-four (9.5%) patients with asthma reported post-COVID-19 syndrome at 3 months. One of them had severe asthma, four moderate, and two mild, four of them from the previously hospitalized group. Two out of these four complained of coughing, one reported dyspnoea, and one fatigue. All but one of the seven patients with post-COVID-19 syndrome received inhaled corticosteroids. Only one patient with post-COVID-19 syndrome was classified as asthma of the non-T phenotype. The authors concluded that, in their experience, patients with preexisting asthma have a lower prevalence of post-COVID-19 syndrome [22]. The reason for this observation could be related to the immune characteristics of the patients or to treatment. In fact, in vitro studies have shown that inhaled glucocorticoids reduce SARS-CoV-2 replication in the airway epithelium [35]. Most of the symptomatic patients with asthma were receiving these drugs.

The largest group of asthma sufferers (*n* = 4500) was surveyed in the UK [33]. The results of the presented study indicated that the COVID-19 group (*n* = 471, 10.5%) reported increased inhaler use and worse asthma management compared to those who did not report COVID-19, but did not differ by gender, ethnicity, or household income. Those with post-COVID-19 syndrome (56,1%) were more likely to describe their breathing as worse or much worse after their initial illness (73.7% vs. 34.8%, *p* < 0.001), increased use of inhalers (67.8% vs. 34.8%, *p* < 0.001), and worse or much worse asthma management (59.6% vs. 25.6%, *p* < 0.001) [33]. Due to the questionnaire nature of the study methodology, these results should be treated with caution, as they are based on subjective feelings of patients, rather than a reliable medical diagnosis, including here objective verification of COVID-19 infections.

**Figure 2:**
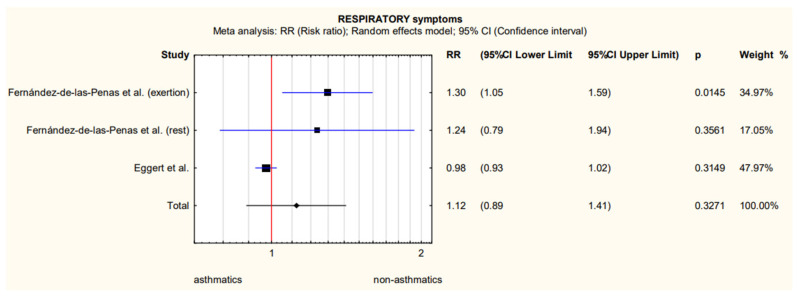
Meta-analytically derived dyspnoea at rest and exertion [26] and cough [32] prevalence in patients with asthma after COVID-19.

The only quantitative data referring to similar outcomes that could be extracted from the two studies concerned respiratory symptoms, i.e., dyspnoea at rest and exertion and cough in 659 patients with asthma [16,22]. These were used to perform a meta-analysis, the results of which are shown in Figure 2 and Table 2. Data on the number of patients experiencing dyspnoea at rest and exertion were obtained from the same study [26]. 

An analysis of the heterogeneity of the results obtained indicated significant heterogeneity (Table 2), and the insufficient number of studies that provide numerical data makes it impossible to perform a sensitivity analysis or to analyse the results in subgroups of more homogeneous populations.

### Methodological Quality of Included Studies

**Figure 3:**
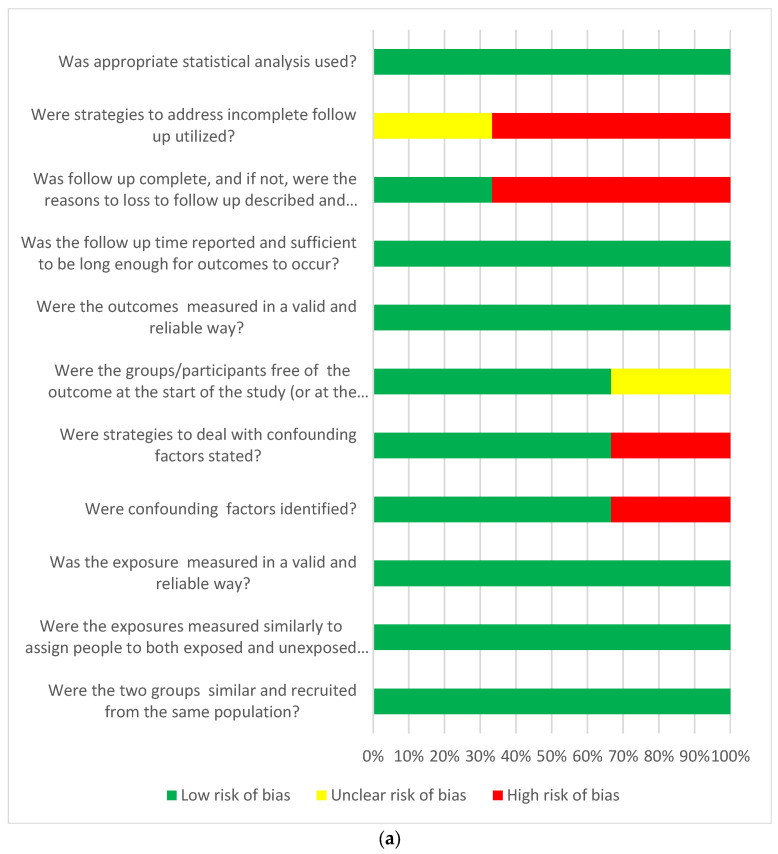

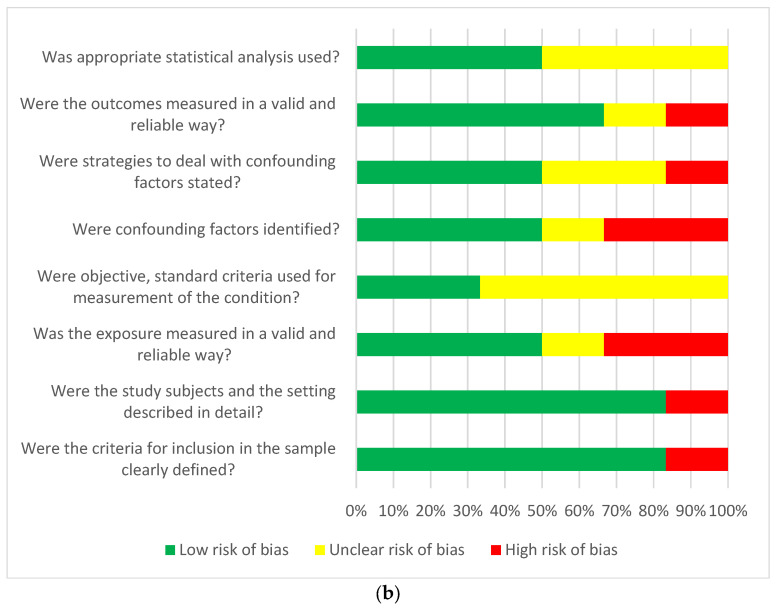
(**a**) Answers to the risk of bias questions presented as percentages across studies included in the systematic review. Cohort studies (*n* = 3) assessment of risk of bias. (**b**) Answers to the risk of bias questions presented as percentages across studies included in the systematic review. Cross-sectional studies (*n* = 6) risk of bias assessment.

Taking into account the risk of bias in the publications that qualified for the final analysis, we used JBI tools intended for the critique or evaluation of research evidence and dedicated to observational cohorts or cross-sectional study. The dominant risk of bias according to the JBI tools was defined as low in most of the assessed aspects of the analysed publications. Only in the aspect of the strategies used in the cohort studies was an unclear or high risk of bias found in the case of incomplete follow-up. Generally, a low risk of bias was found to be higher in cohort studies than in cross-sectional studies (Figure 3a,b).

## 4. Discussion

Many studies have addressed the question of whether asthma among patients with older age, cardiovascular disease, obesity, and diabetes are other risk factors for poor outcomes of COVID-19 [36,37,38]. A recent meta-analysis concluded that preexisting asthma was a predictor of intubation, particularly in young and obese patients with COVID-19 [39]. At the same time, the authors indicated the need for large-scale studies that allow adjustment for confounders such as severity and phenotype of asthma, applied medication, personal factors, and comorbidities to assess the true impact of asthma on susceptibility to and outcome of COVID-19 [32]. In view of the growing interest in chronic and prolonged symptoms of SARS-CoV2 infection, the objective of this review was to examine the frequency and severity of long-term clinical symptoms in the population of asthma patients. 

At the stage of formulating the inclusion criteria, we decided to use only the reports for which we had no doubts about the reliability of the diagnosis, both in terms of asthma and COVID-19. Using an objective and standard criterion for the measurement of the condition is one of the criteria for assessing the reliability of the primary research included in the systematic review and determines the certainty of the formulated conclusions. To remain under restrictive inclusion criteria, we base our conclusions only on five primary studies. Two of them allowed for a quantitative analysis of the risk of having long symptoms more than 12 weeks after the onset of the disease [26,32]. However, the width of 95% CI from the meta-analysis (Figure 2) indicated the lack of statistical power and the resulting significant heterogeneity (Table 2) did not allow us to make a clear statement about the lack of differences between people with and without confirmed asthma diagnosis. The reason for this high heterogeneity may be that the Spanish study observed only people who were hospitalized in the acute phase with COVID-19, and in the US study only 100 people with asthma were hospitalized, the remaining 498 patients with asthma were not. In addition, Spanish researchers checked distant symptoms more than 7 months after hospitalization, and US researchers 90 days after the onset of the disease. 

Of the objectively measured variables, only two papers, indicated in Section 3 of our review, presented the values of the spirometry variables [27,30]. Both publications do not report the patient-level data obtained by the 27 asthma patients selected from the observed cohorts. The univariate regression analysis presented in the first indicated that asthma was not a factor that caused a significant decrease in the recorded values of these spirometry variables [27]. In this study by German researchers, of the nine patients with asthma, four required hospitalization in the severe phase of the disease, of which two were seriously ill and required ventilatory support. Different results in this regard were presented in the second study [30], in which patients with asthma (*n* = 18) had a higher probability of altered DLCO. This cohort was examined 6 months after the onset of COVID-19 when the patients had been treated in the hospital due to clinical/instrumental signs of interstitial pneumonia and acute respiratory failure [30]. This may indicate a potentially higher risk of long-term changes in spirometric variables in patients with asthma with severe COVID-19 infections who require hospitalization and ventilator support. It should be noted that this is consistent with the observations of Garcia Pachon, in which, out of a 74-person group of patients with asthma suffering from COVID-19, seven were diagnosed with post-COVID-19 syndrome, of which up to four required hospitalization in the acute phase of infection. 

Recent data showed that the expression of angiotensin converting enzyme 2 (ACE2) and transmembrane protease serine 2 (TMPRSS2) were decreased with the use of inhaled corticosteroids (ICS). In fact, in vitro studies have shown that inhaled glucocorticoids reduce SARS-CoV-2 replication in the airway epithelia [35], suggesting a possible reason why patients with asthma do not appear to experience some of the most serious and life-threatening manifestations of COVID-19 disease. However, Garcia-Pachon et al. pointed out that all but one of the seven patients with post-COVID-19 syndrome received inhaled corticosteroids [22]. In Foster et al., the rate of asthma exacerbation in patients who took inhaled corticosteroids and long acting beta-agonists (ICS + LBA) (*n* = 25, 53.3%) or ICS alone (*n* = 13, 55.2%) did not differ from those who did not take ICS (*n* = 38, 52.6%), (*p* = 0.82) [31]. 

It seems that a conclusion about regularity and inhaled corticosteroid administration dose that may have an impact on long-term results cannot be reached with certainty. Some authors, such as Mahdavinia et al. who confirmed that asthma was independently associated with a prolonged duration of intubation for coronavirus disease, had adjusted the results of their analyses for the use of albuterol and systemic steroids, but it would be important to know how many patients were being treated with inhaled corticosteroids and the results in these patients [40]. Data from publications that qualified for the review, due to the lack of comparative groups, did not allow us to compare the impact of using various types of anti-asthmatic treatment (steroids, biological treatment) on the occurrence of long-COVID-19 in patients with asthma. Additional research focused on this issue is needed to obtain more information.

On the other hand, recent research results indicate that, for patients with asthma, pre-existing eosinophilia (absolute eosinophil count (AEC) ≥ 150 cells/μL) was protective of COVID-19-associated admission, and the development of eosinophilia (AEC ≥ 150 cells/μL) during hospitalization was associated with a decrease in mortality [41]. It is confirmed by the study included in this review, where of the 74 people with asthma, in 46 patients asthma was classified as allergic, 7 as eosinophilic, and 21 as non-T2. Having a Th2-asthma phenotype could be an important predictor of reduced morbidity and mortality from COVID-19 that should be further explored in prospective studies, but it is very important to realize that, in the study by Gracia-Pachon mentioned above, only one patient with post-COVID-19 syndrome was classified as non-T2 phenotype asthma [22]. The long-term follow-up of numerous and diverse patients with different asthma phenotypes, objective evaluation of their respiratory status, analysis of blood samples, and very detailed data on the form and intensity of drug treatment would be required to further explore that relation. We had hoped to identify such studies while building the assumptions of the review formulated in the protocol. Unfortunately, the nine eligible papers found do not allow such conclusions.

The advantage of the review is a comprehensive search of the available literature on COVID-19 and asthma, together with a detailed evaluation of the identified publications in terms of their methodological quality and risk of bias. In our review we were limited by a small number of eligible studies, significant heterogeneity between them, lack of standard diagnosis of asthma, not reported strategies used to address incomplete follow-up, insufficient identification of confounders, and improper way of exposition measures in some studies. A total of 848 primary publications also did not specify the variety of virus that infected patients with asthma. Subsequent mutations may have had a different effect in these patients. Therefore, drawing conclusions from this systematic review should be approached with caution. 

## 5. Conclusions

Long-term symptoms (lasting at least 12 weeks) caused by SARS-CoV-2 infection among patients with asthma are: lower respiratory symptoms such as cough, dyspnoea during exertion and at rest; moreover: fatigue, memory loss, concentration loss, cognitive blunting—brain fog, gastrointestinal disorders—diarrhoea, tachycardia/palpitations, ocular disorders, ageusia/hypogeusia, anosmia/hyposmia, throat pain, depressive symptoms, anxiety symptoms, poor sleep quality. Some of them reported an increase in the use of reliever inhalers and that their asthma treatment was worse or much worse. 

These symptoms appear to be similar in strength and frequency in patients with and without asthma diagnosis. Establishing the unequivocal differences between post-COVID-19 symptoms in patients with asthma and without asthma was impossible due to inconsistent results of the eligible publications in which the relevant groups of patients were compared. The studies included in this review do not allow conclusions to be drawn about the long-term consequences for asthma symptoms, lung function, intensity of pharmacological treatment, physical fitness, and mental health of adults with asthma after SARS-CoV-2 infection. The data obtained in the review were too sparse also to draw conclusions on the connection between GINA severity category of asthma and long-term consequences of COVID-19. They did not allow for a clear conclusion on whether the degree of asthma control and asthma phenotype prior to COVID-19 infection affects these variables. 

Our findings support the need for clinical follow-up, with a holistic assessment to include symptoms of physical and mental health in that group of patients. 

## Figures and Tables

**Table 1:** Studies of asthma and long-COVID-19 included in the systematic review.

Article [Reference]	Study Type Age	Study Country Study Period	Total Number of Cases *n*=	COVID-19 and Asthma + Patients *n*=	Male *n* = (%)	Female *n* = (%)	COVID-19 Diagnosis	Asthma Diagnosis	Persistent Post-COVID-19 Symptoms, *n* (%)		
Eggert et al. (2022) [32]	Prospective cohort study N/A	USA 1 March–30 September 2020	6976	Hospitalized = 100 Not Hospitalized = 498	COVID−19 Asthma + Hospitalized = 42 (42.0%) Not Hospitalized = 202 (40.6%) COVID−19 Asthma- Hospitalized = 247 (48.9%) Not Hospitalized = 2144 (47.7%)	COVID−19 Asthma + Hospitalized = 58 (58.0%) Not Hospitalized = 296 (59.4%) COVID−19 Asthma-Hospitalized = 258 (51.1%) Not Hospitalized = 2336 (52.0%)	Positive diagnosis by authorized SARS-CoV-2 nucleic acid amplification test from either nasal, nasopharyngeal swab, or bronchoalveolar lavage	Using an institutional informatics platform, we comprehensively characterized a cohort of patients who tested positive for SARSCoV-2 and further categorized them by baseline asthma diagnosis using the International Classification of Disease (ICD-9/10) diagnostic codes and hospitalization status; 24 Patients were categorized as having allergic asthma if they had at least one ICD-10 code for a coexisting allergic disorder the GINA 2020 guidelines	Symptoms at: - 30 days (96.9% in asthmatic and 98.6% in non-asthmatic), - 60 days (84.4% in asthmatic and 89.9% in non-asthmatic), - 90 days (75% in asthmatic and 76.8% in the non-asthmatic). Resolution of lower respiratory symptoms did not differ between asthmatics and non-asthmatics with more than 50% of both groups still reporting lower respiratory symptoms 90 days after initial symptoms.		
Fernández-de-las-Peñas et al. (2021) [26]	Cross-sectional study Age, mean (SD), years Asthmatic: 55 (17) Non-asthmatic: 55 (16.5)	Spain 30 March–31 May 2020	400	Asthmatic (*n* = 61) Non-asthmatic (*n* = 122)	Asthmatic: 15 (24.6%) Non-asthmatic: 30 (24.6%)	Asthmatic: 46 (75.4%) Non-asthmatic: 92 (75.4%)	Positive diagnosis of SARS-CoV-2 by RT-PCR technique and radiological findings	Assessed by the 2019 Global Initiative for Asthma (GINA) guidelines		Asthmatic [%]	Non-asthmatic [%]
									Dyspnoea on exertion	75.4	58.2
									Fatigue	65.6	61.5
									Dyspnoea rest	34.4	27.8
									Memory loss	18	16.4
									Skin rashes	13.1	10.6
									Concentration loss	13.1	11.4
									Cognitive Blunting—Brain fog	9.9	8.2
									Gastrointestinal Disorders Diarrhoea	3.3	4.1
									Tachycardia-palpitations	9.8	6.5
									Ocular Disorders	6.6	7.2
									Ageusia/hypogeusia	1.6	2.5
									Anosmia/hyposmia	6.6	1.6
									Throat pain	3.3	2.5
									Depressive symptoms	27.9	22.1
									Anxiety symptoms	14.75	11.5
									Poor sleep quality	54.1	41.8
Philip et al. (2022) [33]	Cross-sectional study Median age: 50–59 years	UK October 2020	4500	Had COVID-19: 471 Not sure if had COVID-19: 972	All survey respondents: 856 (19.12%) Not sure if had COVID-19: 190 (19.59%) Had COVID-19: 84 (17.99%)	All survey respondents: 3618 (80.79%) Not sure if had COVID-19: 778 (80.21%) Had COVID-19: 382 (81.80%)	Self-reported COVID-19	Self-reported asthma	261 patients with long-COVID-19 (56.13%). Compared with those who had not had long-COVID-19 and those who were not sure, a higher proportion of individuals who reported that they had long COVID-19 described their breathing as worse or much worse (73.7% vs. 34.8%, *p* < 0.001), an increase in reliever inhaler use (67.8% vs. 34.8%, *p* < 0.001), and that their asthma management was worse or much worse (59.6% vs. 25.6, *p* < 0.001).		
Pachon et al. (2021) [22]	Prospective cohort study Age > 14 years	Spain 3 March–11 December 2020	2995	74	32 (43%)	42 (57%)	Positive diagnosis of SARS-CoV-2 by RT-PCR technique	Assessed by international GINA recommendations (https://ginasthma.org/)			*n* [%]
									Post-COVID-19 syndrome at 3 months in patients with asthma		7 (9.5%)
									Dyspnoea		1
									Fatigue		3
									Anosmia/Hyposmia		1
									Cough		2
Munker et al. (2021) [27]	Prospective cohort study The mean age: 49.6 ± 17.4	Germany March–August 2020	76	9	33 (46.1%)	43 (53.9%)	Confirmed SARS-CoV-2 infection (PCR) or positive Anti-SARS-CoV-2 Immunoglobulin G (IgG) titer	NR	Univariate regression analysis for patients with bronchial asthma		
									DLCOcSB		*p* > 0.05.
									TLC		
									FVC		
									FEV1		
Munblit et al. (2021) [28]	Prospective cohort study The median age 56 years (IQR, 46–66; range, 18–100 years)	Russia 8 April–10 July 2020	2649	121	1296 (48.9%)	1353 (51.1%)	Positive diagnosis of SARS-CoV-2 by RT-PCR and/or clinically confirmed infection	NR	Asthma and chronic pulmonary disease were not associated with persistent symptoms overall, but asthma was associated with neurological (1.95, 1.25 to 2.98) and mood and behavioural changes (2.02, 1.24 to 3.18), and chronic pulmonary disease was associated with chronic fatigue (1.68, 1.21 to 2.32).		
Maestre-Muñiz et al. (2021) [29]	Cross-sectional study Mean age (SD): 65.1 years (17.5)	Spain 15 March–10 May 2021	543	39	NR	NR	Laboratory-confirmed COVID-19	Assessed by the Global Initiative for Asthma (GINA)’s treatment steps for adults	Asthma developed during the year after acute COVID-19 recovery (*n* = 2; 0.4%). Patients with asthma alive after one year/at baseline *n* = 39/51 (76.5%). Treatment intensification (*n* = 8, 20.5%).		
Faverio et al. (2021) [30]	Prospective, observational cohort study Median interquartile range age: 61.1 years	Italy March–June 2020	All: 312 Oxygen group:71 CPAP group: 144 IMV group: 97	Asthma in: Oxygen group:9 CPAP group: 4 IMV group: 4	229 (73%)	83 (27%)	Diagnosis of SARS-CoV-2 infection by positive PCR on nasal-pharyngeal swab or on bronchoalveolar lavage in case of double negative nasal-pharyngeal swabs performed at least 24 h apart	N/A	Patients with asthma presented higher odds of altered DLCO (OR = 4.86, 95% CI: 1.09; 21.68); odds of chest X-ray alterations in patients with asthma haven’t been higher (OR = 2.79; 95% CI: 0.58; 13.29).		
Foster et al. (2021) [31]	Prospective cohort study N/A	USA February–April 2020	76	76	NR	NR	N/A	History of asthma	Stratified by asthma severity, 66.6% of subjects with intermittent, 50.0% with mild persistent and 68.4% with moderate/severe persistent asthma experienced exacerbations (*p* = 0.78). The asthma exacerbation rate in patients who took inhaled corticosteroids (ICS+LABA) (*n* = 25, 53.3%) or ICS alone (*n* = 13, 55.2%) did not differ from those who were not taking ICS (*n* = 38, 52.6%), (*p* = 0.82). 42.3% of asthma patients with a history of allergic rhinitis versus 64.5% of nonallergic patients experienced an exacerbation (*p* = 0.086).		

NR, not reported; N/A, not applicable.

**Table 2:** Heterogeneity analysis of studies qualified for the meta-analysis [26,32].

Q	df	*p*	T2	95% CI (T2)	I^2^	95% CI (I^2^)
7.73	2	0.021	0.0280	0.0016–0.1161	74.13%	13.72–92.24%

Q—Cochran’s measure of heterogeneity, df—degree of freedom, *p*—probability value, T2—heterogeneity variance, CI—confidence interval, I^2^—heterogeneity statistic.

## Data Availability

Not applicable.

## Supplementary Materials

The following supporting information can be downloaded at: https://www.mdpi.com/article/10.3390/biomedicines11082268/s1, Table S1: Key words used in electronic search of the MEDLINE/PubMed, EMBASE, Web of Science, CINAHL and Scopus Scholar—publication date from 1 January 2020 to 1 June 2022.

## References

[1] Global Initiative for Asthma GINA Global Strategy for Asthma Management and Prevention Global Initiative for Asthma (GINA) What’s New in GINA 2021?. www.ginasthma.org.

[2] Nassoro D.D., Mujwahuzi L., Mwakyula I.H., Possi M.K., Lyantagaye S.L. (2021). Asthma and COVID-19: Emphasis on Adequate Asthma Control. Can. Respir. J..

[3] Adir Y., Saliba W., Beurnier A., Humbert M. (2021). Asthma and COVID-19: An update. Eur. Respir. Rev..

[4] Wu Z., McGoogan J.M. (2020). Characteristics of and Important Lessons from the Coronavirus Disease 2019 (COVID-19) Outbreak in China: Summary of a Report of 72 314 Cases from the Chinese Center for Disease Control and Prevention. JAMA.

[5] Marcello R.K., Dolle J., Grami S., Adule R., Li Z., Tatem K., Anyaogu C., Apfelroth S., Ayinla R., Boma N. (2020). Characteristics and outcomes of COVID-19 patients in New York City’s public hospital system. PLoS ONE.

[6] Richardson S., Hirsch J.S., Narasimhan M., Crawford J.M., McGinn T., Davidson K.W., the Northwell COVID-19 Research Consortium (2020). Presenting Characteristics, Comorbidities, and Outcomes Among 5700 Patients Hospitalized with COVID-19 in the New York City Area. JAMA.

[7] Caminati M., Lombardi C., Micheletto C., Roca E., Bigni B., Furci F., Girelli D., Senna G., Crisafulli E. (2020). Asthmatic patients in COVID-19 outbreak: Few cases despite many cases. J. Allergy Clin. Immunol..

[8] Beurnier A., Jutant E.-M., Jevnikar M., Boucly A., Pichon J., Preda M., Frank M., Laurent J., Richard C., Monnet X. (2020). Characteristics and outcomes of asthmatic patients with COVID-19 pneumonia who require hospitalisation. Eur. Respir. J..

[9] Laorden D., Domínguez-Ortega J., Carpio C., Barranco P., Villamañán E., Romero D., Quirce D., Alvarez-Sala S., Camperos R., De Agrela I. (2023). Long COVID outcomes in an asthmatic cohort and its implications for asthma control. Respir. Med..

[10] Liu S., Cao Y., Du T., Zhi Y. (2020). Prevalence of Comorbid Asthma and Related Outcomes in COVID-19: A Systematic Review and Meta-Analysis. J. Allergy Clin. Immunol. Pract..

[11] Zhu Z., Hasegawa K., Ma B., Fujiogi M., Camargo C.A., Liang L. (2021). Association of asthma and its genetic predisposition with the risk of severe COVID-19. J. Allergy Clin. Immunol..

[12] Lee B., Lewis G., Agyei-Manu E., Atkins N., Bhattacharyya U., Dozier M., Rostron J., Sheikh A., McQuillan R., Theodoratou E. (2022). Risk of serious COVID-19 outcomes among adults and children with moderate-to-severe asthma: A systematic review and meta-analysis. Eur. Respir. Rev..

[13] Kermani N.Z., Song W.J., Badi Y., Versi A., Guo Y., Sun K., Bhavsar P., Howarth P., Dahlen S.-E., Sterk P. (2021). Sputum ACE2, TMPRSS2 and FURIN gene expression in severe neutrophilic asthma. Respir. Res..

[14] Peters M.C., Fahy J.V. (2020). Erratum: COVID-19–related Genes in Sputum Cells in Asthma: Relationship to Demographic Features and Corticosteroids. Am. J. Respir. Crit. Care Med. Am. Thorac. Soc..

[15] Coronavirus (COVID-19): Long-Term Health Effects. https://www.gov.uk/government/publications/covid-19-long-term-health-effects/covid-19-long-term-health-effects.

[16] Akbarialiabad H., Taghrir M.H., Abdollahi A., Ghahramani N., Kumar M., Paydar S., Razani B., Mwangi J., Asadi-Poota A.A., Malekmakan L. (2021). Long COVID, a comprehensive systematic scoping review. Infection.

[17] Greenhalgh T., Knight M., A’court C., Buxton M., Husain L. (2020). Management of post-acute covid-19 in primary care. BMJ.

[18] Garg P., Arora U., Kumar A., Wig N. (2021). The “post-COVID” syndrome: How deep is the damage?. J. Med. Virol..

[19] Cabrera Martimbianco A.L., Pacheco R.L., Bagattini Â.M., Riera R. (2021). Frequency, signs and symptoms, and criteria adopted for long COVID-19: A systematic review. Int. J. Clin. Pract..

[20] Carvalho-Schneider C., Laurent E., Lemaignen A., Beaufils E., Bourbao-Tournois C., Laribi S., Flament T., Ferreira-Maldent N., Bruyère F., Stefic K. (2021). Follow-up of adults with noncritical COVID-19 two months after symptom onset. Clin. Microbiol. Infect..

[21] Chiner-Vives E., Cordovilla-Pérez R., de la Rosa-Carrillo D., García-Clemente M., Izquierdo-Alonso J.L., Otero-Candelera R., Pérez-de Llano L., Sellares-Torres J., de Granda-Orive J.I. (2022). Short and Long-Term Impact of COVID-19 Infection on Previous Respiratory Diseases. Arch. Bronconeumol..

[22] Garcia-Pachon E., Grau-Delgado J., Soler-Sempere M.J., Zamora-Molina L., Baeza-Martinez C., Ruiz-Alcaraz S., Padilla-Navas I. (2021). Low prev-alence of Covid-19 syndrome in patients with asthma. J. Infect..

[23] Adeloye D., Elneima O., Daines L., Poinasamy K., Quint J.K., Walker S., E Brightling C., Siddiqui S., Hurst J.R., Chalmers J.D. (2021). The long-term sequelae of COVID-19: An international consensus on research priorities for patients with pre-existing and new-onset airways disease. Lancet Respir. Med..

[24] Covidence. https://www.covidence.org/.

[25] Critical Appraisal Tools. https://jbi.global/critical-appraisal-tools.

[26] Fernández-De-Las-Peñas C., Palacios-Ceña D., Gómez-Mayordomo V., Rodríuez-Jiménez J., Palacios-Ceña M., Velasco-Arribas M., Guijarro C., I De-La-Llave-Rincón A., Fuensalida-Novo S., Elvira-Martínez C.M. (2021). Long-term post-COVID symptoms and associated risk factors in previously hospitalized patients: A multicenter study. J. Infect..

[27] Munker D., Veit T., Barton J., Mertsch P., Mümmler C., Osterman A., Khatamzas E., Barnikel M., Hellmuth J.C., Münchhoff M. (2022). Pulmonary function impairment of asymptomatic and persistently symptomatic patients 4 months after COVID-19 according to disease severity. Infection.

[28] Munblit D., Bobkova P., Spiridonova E., Shikhaleva A., Gamirova A., Blyuss O., Nekliudov N., Bugaeva P., Andreeva M., DunnGalvin A. (2021). Incidence and risk factors for persistent symptoms in adults previously hospitalized for COVID-19. Clin. Exp. Allergy.

[29] Maestre-Muñiz M.M., Arias Á., Mata-Vázquez E., Martín-Toledano M., López-Larramona G., Ruiz-Chicote A.M., Lucendo A.J. (2021). Long-term outcomes of patients with coronavirus disease 2019 at one year after hospital discharge. J. Clin. Med..

[30] Faverio P., Luppi F., Rebora P., Busnelli S., Stainer A., Catalano M., Pesci A. (2021). Supplementary Material for: Six-Month Pulmonary Impairment after Severe COVID-19: A Prospective, Multicentre Follow-Up Study. Respiration.

[31] Foster K., Moore D., Jauregui E., Andy-Nweye A., Mahdavinia M. (2021). Five-month Outcomes for Asthmatics with COVID-19 and Associations with Atopy and Inhaled Corticosteroids Use. J. Allergy Clin. Immunol..

[32] Eggert L.E., He Z., Collins W., Lee A.S., Dhondalay G., Jiang S.Y., Fitzpatrick J., Snow T.T., Pinsky B.A., Artandi M. (2022). Asthma phenotypes, associated comorbidities, and long-term symptoms in COVID-19. Allergy Eur. J. Allergy Clin. Immunol..

[33] Philip K.E.J., Buttery S., Williams P., Vijayakumar B., Tonkin J., Cumella A., Renwick L., Ogden L., Quint J.K., Johnston S.L. (2022). Impact of COVID-19 on people with asthma: A mixed methods analysis from a UK wide survey. BMJ Open Respir. Res..

[34] Coronavirus (COVID-19) Infection Survey, Antibody and Vaccination Data for the UK: 13 May 2021. www.ons.gov.uk/peoplepopulationandcommunity/healthandsocialcare/conditionsanddiseases/articles/coronaviruscovid19infectionsurveyantibodydatafortheuk/13may2021.

[35] Matsuyama S., Kawase M., Nao N., Shirato K., Ujike M., Kamitani W., Fukushi S. (2020). The Inhaled Steroid Ciclesonide Blocks SARS-CoV-2 RNA Replication by Targeting the Viral Replication-Transcription Complex in Cultured Cells. J. Virol..

[36] Choi Y.J., Park J.Y., Lee H.S., Suh J., Song J.Y., Byun M.K., Park H.J. (2021). Effect of asthma and asthma medication on the prognosis of patients with COVID-19. Eur. Respir. J..

[37] Green I., Merzon E., Vinker S., Golan-Cohen A., Magen E. (2021). COVID-19 Susceptibility in Bronchial Asthma. J. Allergy Clin. Immunol. Pract..

[38] Izquierdo J.L., Almonacid C., González Y., Del Rio-Bermudez C., Ancochea J., Cárdenas R., Lumbreras S., Soriano J.B. (2020). The impact of COVID-19 on patients with asthma. Eur. Respir. J..

[39] Hussein M.H., Elshazli R.M., Attia A.S., Nguyen T.P., Aboueisha M., Munshi R., Toraih E.A., Fawzy M.S., Kandil E. (2020). Asthma and COVID-19; different entities, same outcome: A meta-analysis of 107,983 patients. J. Asthma.

[40] Mahdavinia M., Foster K.J., Jauregui E., Moore D., Adnan D., Andy-Nweye A.B., Bishehsari F. (2020). Clinical Communications Asthma prolongs intubation in COVID-19. J. Allergy Clin. Immunol. Pract..

[41] Ferastraoaru D., Hudes G., Jerschow E., Jariwala S., Karagic M., de Vos G., Rosenstreich D., Ramesh M. (2021). Eosinophilia in Asthma Patients Is Protective Against Severe COVID-19 Illness. J. Allergy Clin. Immunol. Pract..

