# Supplementary material for: Post-COVID-19 Symptoms in Adults with Asthma—Systematic Review
Source: Biomedicines. 2023 Aug 14;11(8):2268. doi: 10.3390/biomedicines11082268 (PMC10452668; doi:10.3390/biomedicines11082268)
Supplement: Supplementary file 1 [file biomedicines-11-02268-s001.zip › biomedicines-2488548-supplementary.pdf]

Supplementary Materials

Table S1. Key words used in electronic search of the MEDLINE/PubMed, EMBASE, Web of Science, CINAHL and Scopus Scholar—publication date from 1 January 2020 to 1 June 2022.

	**COVID Terms**
1	SARS-CoV-2
2	COVID-19
3	COVID
4	coronavirus
5	post-COVID-19
6	post-COVID
7	long-COVID-19
8	long-COVID
9	1 OR 2 OR 3 OR 4 OR 5 OR 6 OR 7 OR 8
	**Length Terms**
10	Long
11	Late
12	Persistent
13	Post
14	Chronic
15	10 OR 11 OR 12 OR 13 OR 14
	**Additional Terms**
16	symptoms
17	complications
18	consequences
19	16 OR 17 OR 18
20	asthma
	**Synthesis**
21	9 AND 15 AND 19 AND 20
